# Global Distribution of Human Protoparvoviruses

**DOI:** 10.3201/eid2407.172128

**Published:** 2018-07

**Authors:** Elina Väisänen, Ushanandini Mohanraj, Paula M. Kinnunen, Pikka Jokelainen, Haider Al-Hello, Ali M. Barakat, Mohammadreza Sadeghi, Farid A. Jalilian, Amir Majlesi, Moses Masika, Dufton Mwaengo, Omu Anzala, Eric Delwart, Olli Vapalahti, Klaus Hedman, Maria Söderlund-Venermo

**Affiliations:** University of Helsinki, Helsinki, Finland (E. Väisänen, U. Mohanraj, P.M. Kinnunen, P. Jokelainen, O. Vapalahti, K. Hedman, M. Söderlund-Venermo);; Statens Serum Institut, Copenhagen, Denmark (P. Jokelainen);; Estonian University of Life Sciences, Tartu, Estonia (P. Jokelainen);; National Institute for Health and Welfare, Helsinki (H. Al-Hello); Al-Hussein Teaching Hospital, Thi-Qar Governorate, Iraq (A.M. Barakat);; Blood Systems Research Institute, San Francisco, California, USA (M. Sadeghi, E. Delwart);; University of California, San Francisco (M. Sadeghi, E. Delwart);; Hamadan University of Medical Sciences, Hamadan, Iran (F.A. Jalilian, A. Majlesi);; University of Nairobi, Nairobi, Kenya (M. Masika, D. Mwaengo, O. Anzala);; Helsinki University Hospital, Helsinki (O. Vapalahti, K. Hedman)

**Keywords:** human protoparvovirus, viruses, parvovirus, parvoviridae, bufavirus, tusavirus, cutavirus, cutaneous T-cell lymphoma, gastroenteritis, serologic analysis, epidemiology, global distribution, enteric infections

## Abstract

Development of next-generation sequencing and metagenomics has revolutionized detection of novel viruses. Among these viruses are 3 human protoparvoviruses: bufavirus, tusavirus, and cutavirus. These viruses have been detected in feces of children with diarrhea. In addition, cutavirus has been detected in skin biopsy specimens of cutaneous T-cell lymphoma patients in France and in 1 melanoma patient in Denmark. We studied seroprevalences of IgG against bufavirus, tusavirus, and cutavirus in various populations (n = 840), and found a striking geographic difference in prevalence of bufavirus IgG. Although prevalence was low in adult populations in Finland (1.9%) and the United States (3.6%), bufavirus IgG was highly prevalent in populations in Iraq (84.8%), Iran (56.1%), and Kenya (72.3%). Conversely, cutavirus IgG showed evenly low prevalences (0%–5.6%) in all cohorts, and tusavirus IgG was not detected. These results provide new insights on the global distribution and endemic areas of protoparvoviruses.

Parvoviruses are small, nonenveloped, single-stranded DNA viruses that infect a wide variety of animals ranging from insects and shrimp to birds and mammals. Human parvoviruses belong to 4 genera: *Erythroparvovirus*, *Bocaparvovirus*, *Tetraparvovirus*, and *Dependoparvovirus* (*1*). The recently described bufavirus, tusavirus, and cutavirus are the first members of the genus *Protoparvovirus* found in humans. All 3 viruses were identified by next-generation sequencing and metagenomics in feces of children with diarrhea: bufavirus from Burkina Faso in 2012, tusavirus from Tunisia in 2014, and cutavirus from Brazil and Botswana in 2016 (*2**–**4*). In addition, cutavirus was detected by in silico analysis of existing next-generation sequencing libraries and by PCR of malignant skin tissues of patients in France with cutaneous T-cell lymphoma (*4*).

To date, 3 genotypes of bufavirus have been detected, and bufavirus DNA has been detected in 1 nasal swab specimen of a child in Finland and in <4% of fecal samples from patients with diarrhea in Africa, Europe, and Asia (*2**,**5**–**13*). Recently, a bufavirus 3 sequence was reported in a fecal sample in Peru, which expanded the geographic locations where bufavirus has been detected (*14*). All of these studies have reported DNA sequences of either bufavirus 1 or 3; bufavirus 2 DNA has been detected only in 1 child in Burkina Faso. Humans have been shown to have IgG against all 3 bufavirus genotypes, which also seem to represent distinct serotypes (*12*).

Although the seroprevalence of bufavirus in Finland was found to be low (3.1% in children and in adults born in Finland), the presence of bufavirus IgG in 5/12 adults originating from Asia suggested that the bufavirus prevalence might be higher in other continents. Furthermore, the strong IgG responses indicate that these 3 viruses might cause systemic infections similar to other known human parvoviruses, such as human parvovirus B19, human bocaviruses, and human parvovirus 4 (*15*). In addition to bufavirus in humans, several animal species, including nonhuman primates, shrews, bats, rats, swine, and fur seals, have been shown to be infected with specific bufavirus-like viruses (*16**–**23*).

Conversely, tusavirus DNA has been detected only in feces of 1 child in Tunisia (*3*). In addition, 1 of 228 children in Finland showed a low-level IgG response (*12*). However, these findings are scarce, and more studies are needed to determine whether tusavirus is truly a human virus.

Cutavirus is the newest member of the human parvoviruses. This virus was originally detected in feces of children with diarrhea in 2016; cutavirus DNA was also detected in cancerous tissues of 4/17 patients in France with cutaneous T-cell lymphoma (*4*). Although test results for other skin cancer types and healthy skin examined in this study were negative for cutavirus, 1 melanoma patient in Denmark was shown to have cutavirus DNA in malignant skin (*24*).

The etiologic roles of bufavirus, tusavirus, and cutavirus in human disease remain uncertain and more studies are needed. In this study, we developed a new cutavirus IgG enzyme immunoassay (EIA) and combined it with our existing IgG EIA panel of bufavirus 1–3 and tusavirus. We then analyzed 6 human populations on 4 continents for IgG against these 3 protoparvoviruses. We included veterinarians from Finland (n = 324) to assess the possible contribution of human–animal contact; adults from the United States (n = 84), Iraq (n = 99), and Iran (n = 107); and adults (n = 119) and children (n = 107) from Kenya to identify age-related and geographic distributions of these emerging viruses in humans.

## Materials and Methods

### Study Cohorts

The cohorts included in the study were from 5 countries on 4 continents. The study and all sampling were conducted in accordance with relevant guidelines and regulations.

For the veterinary cohort from Finland, we obtained serum samples from 324 healthy adult volunteers (Table 1). Samples were collected from participants at the national Annual Veterinary Congress in 2009 in Helsinki, Finland (*25*). Most (82%) of the volunteers were veterinarians, veterinary students, or veterinary nurses, and 92% completed an electronic questionnaire to obtain background information. Written informed consent was obtained from all study participants, and the study was approved by the Ethics Committee of Helsinki University Central Hospital.

**Table 1:** Characteristics of cohorts used in study of global distribution of human protoparvoviruses*

Cohort	No. persons	Health status	Mean age, y (range)	No. (%) male:female; unknown	Time of sample collection	Other features
Finland	324	Constitutionally healthy	40.2 (19–79)	45 (13.9):279 (86.1)	2009 Oct	Adults: veterinarians
United States	84	Constitutionally healthy	41.3 (18–72)	64 (76.2):20 (23.8)	2009 Apr	Adults: blood donors
Iraq	99	Constitutionally healthy	39.7 (18–60)	71 (71.4):28 (28.3)	2013 Nov–Dec	Adults: medical staff, blood donors, and university students
Iran	107	Constitutionally healthy	42.2 (18–77)	50 (46.7):57 (53.3)	2015–2016	Adults: blood donors
Kenya, children	107	Febrile at time of sampling (mean temperature 38.6°C, range 37.5°C–40.4°C)	6.9 (0.5–17.8)	59 (55.1):43 (40.2); 5 (4.7)†	2016 Apr–Aug	Children: includes 9 HIV+ (8 receiving HAART)
Kenya, adults	119	Febrile at time of sampling (mean temperature 38.9°C, range 37.5°C–39.8°C)	43.3 (18.2–88.3)	42 (35.3):76 (63.9); 1 (0.8)†	2016 Apr–Nov	Adults: includes 38 HIV+ (35 receiving HAART)

*HAART, highly active antiretroviral therapy; +, positive. †Sex was not specified in the questionnaire.

For the cohort from the United States, we obtained serum samples from 84 healthy blood donors at Blood Systems Research Institute (San Francisco, CA, USA) (Table 1). The samples were collected during April 2009 in 2 locations (Arizona [n = 40] and Mississippi [n = 44]). Under US human and health service regulations, the study of preexisting, deidentified samples is not classified as human subject research.

For the cohort from Iraq, we obtained serum samples from 99 healthy adults (Table 1) to assess exposure of the population to various virus infections in Nasiriyah, Dhi Qar, in southern Iraq (*26*). Written informed consent was obtained from all study participants, and the study was approved by the Ethics Committees of Medical Sciences at Basrah University and the Al-Hussein Teaching Hospital.

For the cohort from Iran, we obtained serum samples from 107 healthy adults (Table 1) at the Hamadan Blood Transfusion Organization (Hamadan, Iran). Informed consent was waived for analysis of these deidentified blood donor samples, and the study was approved by the Ethics Committee of Hamadan University of Medical Sciences.

For the cohort from Kenya, we obtained serum samples from 107 children and 119 adults who had a febrile illness of unknown cause and had visited health clinics in Mwatate, Voi, or Wundanyi in Taita Taveta County in southern Kenya (Table 1). A questionnaire to obtain background information and symptoms was completed by all patients, and written informed consent was obtained from all study participants or guardians of children. The study was approved by the Kenyatta National Hospital–University of Nairobi Ethics and Research Committee.

### Serologic Analysis

#### Cutavirus Capsid Protein 2 Virus-Like Particles

To analyze serum samples for IgG against all human protoparvoviruses, we included cutavirus in an in-house EIA panel for bufavirus genotypes 1–3 and tusavirus (*12*). The virus capsid protein 2 (VP2) gene for cutavirus was cloned from the original DNA extract from feces of the cutavirus DNA–positive child from Brazil (Br337) (*4*) by using primers VP2 fwd Br337 *Bam*HI (5′-TAggatccATGTCAGAACCAGCTAATGATAC-3′) and VP2 rev Br337 *Sal*I (5′-CTCgtcgacTTACAATGTGTAGTTTGGTAGACA-3′) (restriction sites are indicated by lowercase letters).

The obtained VP2 (GenBank accession no MH127919) was used to create a recombinant baculovirus with the Bac-to-Bac system (Invitrogen, Carlsbad, CA, USA), according to the manufacturer’s instructions, and cutavirus VP2 virus-like particles (VLPs) were expressed and purified as described for bufavirus VP2 VLPs and tusavirus VP2 VLPs (*12*). 

#### Combined Bufavirus 1–3/Tusavirus–Cutavirus IgG EIA

We analyzed all serum samples by using the combined bufavirus 1–3/tusavirus–cutavirus IgG EIAs, with insect cell lysate as a control antigen, as described, but included cutavirus VLP antigen in separate wells (*12*). In brief, we applied biotinylated antigens (VP2 VLPs, 80 ng/well) or cell lysate control to streptavidin-coated plates. After incubation and postcoating, we applied serum diluted 1:200 to each well. To detect bound IgG, we used horseradish peroxidase–conjugated antihuman IgG as the secondary antibody and 3,3′,5,5′-tetramethylbenzidine (Dako, Santa Clara, CA, USA) as the substrate.

We measured optical densities (ODs) at 450 nm (Multiskan EX; Thermo Fischer Scientific, Pittsburgh, PA, USA) and subtracted blank ODs from test ODs to get the final OD. We confirmed all samples with an OD >0.1 by using a competition assay, as described (*12**,**27*). In the competition assay, serum antibodies were blocked separately with 3 unbiotinylated antigens in solution: the same (homologous) antigen as in the EIA, the heterologous antigen of the phylogenetically closest protoparvovirus, and the heterologous antigen of a more distant protoparvovirus, before repeating the EIAs. A sample was considered IgG positive when full homologous blocking but no (or partial) heterologous blocking occurred, as described (*12*).

### Statistical Analysis

We performed statistical analysis by using 2 × 2 tables and test statistics (mid p-exact value) in OpenEpi software (https://www.OpenEpi.com). A 2-tailed p value <0.05 was considered statistically significant.

## Results

### Cutavirus IgG EIA and Cross-Reactivity of IgG

**Figure 1:**
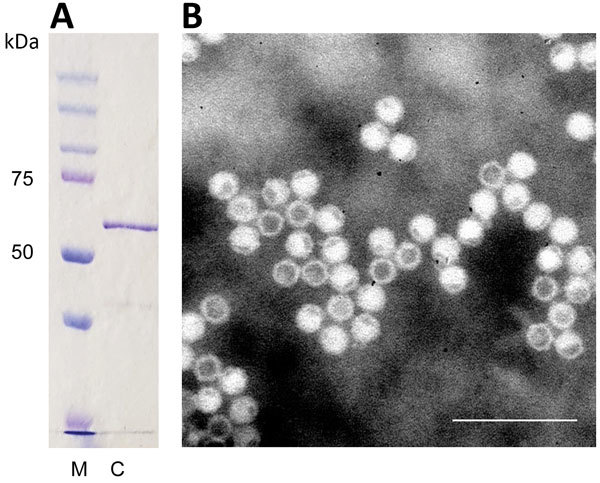
Identification of cutavirus from human serum samples. A) Sodium dodecyl sulfate–polyacrylamide gel electrophoresis of virus capsid protein 2. Lane M, protein size marker; lane C, cutavirus. B) Electron micrograph of cutavirus virus-like particles. Scale bar indicates 100 nm.

Sodium dodecyl sulfate–polyacrylamide gel electrophoresis identified cutavirus VP2s of expected size (≈64 kDa) (Figure 1, panel A). Electron microscopy identified parvovirus-like VLPs with a diameter of ≈25 nm (Figure 1, panel B).

In the cutavirus IgG EIA, it was evident that bufavirus 2 and cutavirus cross-react: in all samples with an OD >0.5 for bufavirus 2 or cutavirus, a reaction was always observed with the other antigen. In samples showing weak reactivity (OD 0.1–0.5) in cutavirus or bufavirus 2 IgG EIAs, both cross-reactivities and single-specific reactivities were observed. In the competition assay, the specific reactivity of bufavirus or cutavirus was blocked completely only by homologous antigen, whereas cross-reactive reactions were blocked by homologous and heterologous antigens. In several instances, heterologous antigen slightly reduced the specific EIA reactivity; however, this reduction was much less than that caused by homologous antigen.

In cohorts from the Middle East and Africa that showed high prevalences of bufavirus IgG, some cross-reactivity was also observed among the 3 bufavirus genotypes, mostly for samples with higher ODs. However, the competition assay used for all positive samples could distinguish genotype-specific reactivity for correct interpretation. Tusavirus IgG did not cross-react with bufavirus IgGs or cutavirus IgG.

### Bufavirus IgG in Adults

Bufavirus IgG was rare among veterinarians from Finland and blood donors from the United States: only 1.9% of the veterinarians and 3.6% of the blood donors had bufavirus IgG (Table 2). In these cohorts, each bufavirus IgG–positive person had antibodies against only 1 bufavirus genotype. No indications of specific animal contact being associated with bufavirus seropositivity were found when we compared background information for bufavirus IgG–positive and bufavirus IgG–negative veterinarians. For blood donors from the United States, all 3 bufavirus IgG–positive samples were from Mississippi. However, all samples from Arizona were negative for bufavirus IgG. The most commonly detected genotype was bufavirus 1 in Finland and bufavirus 3 in the United States (Table 2).

**Table 2:** Seroprevalence of IgG against protoparvoviruses in different population cohorts*

Cohort	No. persons	Any bufavirus IgG†	IgG against bufavirus genotypes			Tusavirus IgG	Cutavirus IgG‡
			1	2‡	3		
Finland, healthy adults (veterinarians)	324	6 (1.9)	4 (1.2)	1 (0.3)§	1 (0.3)	0	16 (4.9)§
United States, healthy adults	84	3 (3.6)	0	0	3 (3.6)	0	0
Iraq, healthy adults	99	84 (84.8)	80 (80.8)	33 (33.3)¶	15 (15.2)	0	1 (1.0)¶
Iran, healthy adults	107	60 (56.1)	55 (51.4)	17 (17.9)	6 (5.6)	0	6 (5.6)
Kenya, febrile children <18 y of age	107	22 (20.6)	3 (2.8)	4 (3.7)	20 (18.7)	0	2 (1.9)
Kenya, febrile adults	119	86 (72.3)	31 (26.1)	43 (36.1)#	56 (47.1)	0	5 (4.2)#

*Values are no. (%) unless otherwise noted.. †Includes persons that were IgG+ against >1 bufavirus genotypes. ‡Unclear bufavirus 2 and cutavirus blocking results were observed for 1§ (0.3%), 2¶ (2.0%), and 3# (2.5%) persons. These values are not included in overall seroprevalence calculations.

In striking contrast to adults from the United States and Finland, including our previous results for students and staff members from Finland (*12*), bufavirus IgG was common in Iraq, Iran, and Kenya, for which 84.8%, 56.1%, and 72.3%, respectively, of adult populations had IgG against >1 bufavirus genotypes (Table 2). In the Middle East, bufavirus 1 was the most common type, whereas in Kenya, bufavirus 3 was the predominant genotype (Table 2). Bufavirus 2 was the second most prevalent bufavirus in all 3 high-prevalence countries. In Iraq, we found that 30 (30.3%) of 99 persons had antibodies against 2 bufavirus genotypes, and 7 (7.0%) of 99 persons had antibodies against all 3 bufavirus genotypes. In adults from Kenya, we found similar prevalences: 30 (25.2%) of 119 persons had antibodies against 2 bufavirus genotypes, and 6 (5.0%) of 119 persons had antibodies against all 3 bufavirus genotypes. However, in Iran, we found that double or triple prevalences were lower: 14 (13.1%) of 107 persons had antibodies against 2 bufavirus genotypes, and 2 (1.9%) of 107 persons had antibodies against all 3 bufavirus genotypes.

In Kenya, HIV-positive patients had a similar bufavirus IgG prevalence as the rest of the cohort: 78.9% (30/38) in HIV-positive adults (mean age 46.3 years, range 27–85 years) vs. 69.1% (56/81) in HIV-negative adults (mean age 41.9 years, range 18–88 years) (p = 0.275). When we compared only the 38 HIV-positive persons and 60 HIV-negative persons within the same age range (27–85 years), bufavirus seroprevalences were even more similar: 79% for HIV-positive persons and 75% for HIV-negative persons (p = 0.669). However, possible undiagnosed cases of infection with HIV and unequal numbers could affect the accuracy of this comparison.

When the adult cohorts were analyzed more closely and persons were divided by age into equal-sized groups of <40 years of age and >40 years of age, we found that for younger adults in Iran, bufavirus seroprevalence was lower than that for older adults (21/54 [38.9%] vs. 39/53 [73.6%]; p = 0.0003). However, a similar distinction was not observed for adults from Iraq or Kenya (Table 3). For veterinarians from Finland and adults from the United States, we found that younger adults also had a lower bufavirus seroprevalence, albeit without statistical power, because of the low overall prevalence of bufavirus IgG in these countries. When we divided the cohorts into persons <30 years of age and >30 years of age, a similar trend was also observed for adults in Kenya (14/35 [56.0%] vs. 70/92 [76.1%]; p = 0.0595). This trend was not observed for persons in Iraq.

**Table 3:** Seroprevalence of bufavirus IgG in adult cohorts, by age, in study of global distribution of human protoparvoviruses*

Cohort	Age group, y	No. persons	No. (%) bufavirus IgG+ (95% CI)	p value	Mean age, y (range)
Finland	<40	165	1 (0.6) (0.0–3.7)	0.096	31.6 (19–39)
	>40	149	5 (3.4) (1.2–7.8)		49.7 (40–79)
United States	<40	38	1 (2.6) (0.0–14.7)	0.731	26.4 (18–39)
	>40	46	2 (4.3) (0.4–15.3)		53.5 (40–72)
Iraq	<40	45	40 (88.9) (76.1–95.6)	0.325	30.8 (18–39)
	>40	54	44 (81.5) (69.0–89.8)		47.2 (40–60)
Iran	<40	54	21 (38.9) (27.0–52.2)	0.0003	30.1 (18–39)
	>40	53	39 (73.6) (60.3–83.7)		55.0 (40–77)
Kenya	<40	52	37 (71.2) (57.7–81.8)	0.890	29.5 (18–39)
	>40	65	47 (72.3) (60.4–81.8)		54.3 (40–88)

*The exact age of 2 adults from Kenya and 10 veterinarians from Finland were not known and they were excluded from the analysis. However, the 2 Kenyans were defined as adults in the overall prevalence calculations on the basis of education and marital status in the questionnaire. All veterinarians were defined as adults on the basis of work-related sample collection site. +, positive.

### Bufavirus IgG in Children in Kenya

**Figure 2:**
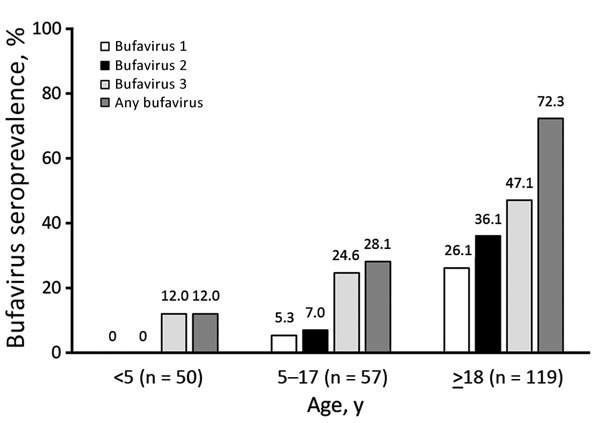
Seroprevalence of bufavirus in Kenya, by age. Several persons (mostly adults) had IgG against >1 bufavirus genotypes; such persons are counted as 1 person in the bufavirus column. Differences in overall bufavirus seroprevalences were statistically significant between younger children vs. older children (p = 0.04345), younger children vs. adults (p<0.000001), and older children vs. adults (p<0.000001).

In Kenya, the bufavirus IgG prevalence in children was significantly lower than that in adults (20.6% in children <18 years of age vs. 72.3% in adults; p<0.0001), but we observed similar proportions of bufavirus genotypes and a predominance of bufavirus 3 in both adults and children. (Table 2). When we divided the cohort of children into those <5 years of age and those 5–17 years of age, the prevalence of bufavirus IgG by age increased from 12% to 28.1% (Figure 2).

### Cutavirus and Tusavirus IgG in Adults and Children

The prevalence of cutavirus IgG was generally low for all groups, ranging from 1.0% in Iraq to 5.6% in Iran, and cutavirus IgG was not detected in adults in the United States (Table 2). In veterinarians in Finland, cutavirus IgG (4.9%) was more common than bufavirus IgG (1.9%) (p = 0.032). Two adults from Kenya and 1 veterinarian from Finland had both cutavirus IgG and bufavirus 2 IgG in their samples, which showed that these 2 antigenically similar viruses can infect the same person and elicit specific immune responses against each virus. However, for 6 patients (1 in Finland, 2 in Iraq, and 3 in Kenya), we could not determine whether the reactivity detected was specific for bufavirus 2, cutavirus, or both. These results were not included in the final prevalence calculations (Table 2). Tusavirus IgG was not detected in any cohort (Table 2).

## Discussion

During the current decade, several new parvoviruses have been detected, mostly because of the development of NGS methods. Bufavirus, tusavirus, and cutavirus are the newest of these viruses detected in human samples (*2**–**4*). Bufavirus has been associated with gastroenteritis, and cutavirus is being studied for its relationship to skin cancers (*4**,**8**–**10**,**12**,**24*). However, studies that attempted to detect bufavirus, tusavirus, and cutavirus DNA in any sample type or virus antibodies in serum samples have been infrequent (*13*).

We found high (50%–85%) seroprevalences of bufavirus IgG in cohorts from the Middle East and Africa, which indicated that bufavirus infections are endemic to these areas. The observed low (1.9%) seroprevalence in veterinarians in Finland is consistent with our previous results for staff members and medical students born in Finland (3.1%) (*12*). The seroprevalence of bufavirus in the United States was similar to that in Finland, although the major genotype was different. In contrast to the diverse epidemiology of antibodies against bufavirus, antibodies against cutavirus appeared globally and were much more evenly distributed and showed a low prevalence. These results provide new insights on the global distribution and identify areas to which protoparvoviruses are endemic.

Because the difference in seroprevalence between persons born in Finland and staff born in Asia in our previous study could also be caused by more frequent animal contacts for 5 persons from Asia (*12*), we included veterinarians in this current study. However, no specific animal contacts for veterinarians from Finland were associated with bufavirus IgG or cutavirus IgG seropositivity. Although species jumps have occurred within protoparvoviruses (*28*), this result is consistent with the general rule of host-order specificity of parvoviruses (*1*). No animal contact information was available for persons from the Middle East, Kenya, or the United States.

The age group results from Kenya, which showed continuously increasing seroprevalences of all 3 bufavirus genotypes, also showed that bufaviruses infect persons of all ages. The lower seroprevalence in children <5 years of age than in older children and adults indicates that the age of acquisition of bufavirus greatly differs from that of human bocavirus 1 (*27*) but resembles that for human parvovirus B19. Also for persons 5–17 years of age, an age-dependent increase was evident, but statistical power was insufficient to further divide these children into narrower age groups. In adults, the age-dependent increase in seroprevalence was detectable in Iran, but not in Kenya or Iraq. This difference could be caused by decreased bufavirus circulation in Iran during the past 30–40 years or to socioeconomic or cultural changes over time. In Finland and the United States, age group prevalences were similar, albeit at low levels.

HIV-positive adults in Kenya did not have a higher seroprevalence of bufavirus IgG than HIV-negative adults. Bufavirus infection route(s) could therefore be hypothesized to differ from those for HIV infection.

The predominant bufavirus genotype (1 or 3) varied between countries studied. Bufavirus 2 was the second most common genotype in the 3 high-prevalence countries (Iraq, Iran, and Kenya). In sharp contrast, bufavirus 2 DNA has hitherto been found in the fecal sample of only 1 child in Burkina Faso, whereas all other bufavirus DNA–positive samples had genotypes 1 or 3 (*2**,**5**,**6**,**8**–**11**,**14*). Our serologic data indicate that bufavirus 2 infections exist and are common in certain areas. Further studies of patients with primary infection should elucidate whether sample type(s) most suitable for detection of bufavirus 2, and also for genotypes 1 and 3, is stool or another type of sample.

In humans, IgG is induced against all 3 bufavirus genotypes and cutavirus, and immune reactions appear to be strong. In our previous report on bufavirus IgG, the 3 bufavirus genotypes were shown to have no mutual cross-reactivity (*12*). In this study, some cross-reactivity was observed between the 3 bufavirus genotypes, particularly among high OD samples in high-seroprevalence cohorts. In addition, cutavirus and bufavirus 2 cross-reactivity was common, which is consistent with the fact that amino acid identities are high within the VP2 gene (82% identity for the amino acid sequence). However, both genotype and species cross-reactivities could be distinguished from specific reactivity in the competition assay, similarly to what is shown for the 4 human bocaviruses (*28*).

Despite some VLP cross-reactivity in the EIA, the 3 bufavirus genotypes do not appear to be cross-protective. Several persons had antibodies against 2 or even 3 protoparvoviruses. Whether the previously formed antibodies against the first virus protects the human host against possible symptoms of the second related virus infection, or worsen the symptoms through antibody-dependent enhancement, is not known. However, it appears that immunity toward different protoparvoviruses does not hamper a sequential infection by heterologous virus (bufavirus genotype or cutavirus) or formation of specific antibodies toward this virus, which is in contrast to the phenomenon of original antigenic sin seen among human bocaviruses (*27**,**29*). Longitudinal studies are needed to assess antibody and protection patterns, both during acute primary infections and during subsequent infections by the other human protoparvoviruses. It will be useful to determine the clinical pictures in these contexts, whether these viruses cause similar primary symptoms and illnesses, and whether they have the same or different tissue tropisms.

We did not detect tusavirus IgG in any cohort. This finding is consistent with results of 2 previous human studies, which reported no or infrequent evidence of tusavirus (*3**,**12*). In addition, a study has reported sequences with some resemblance to tusavirus: a metagenomic analysis of fur seals in Brazil described partial sequences with 39%–82% amino acid similarity to tusavirus (*23*). Further studies on tusavirus DNA or antibodies are needed to determine whether tusavirus is a human or an animal parvovirus whose original detection in human feces was caused by consumption of meat or other products of a tusavirus-infected animal.

In conclusion, we observed major differences in seroprevalence of bufavirus when we compared Finland and the United States with the Middle East and Kenya. The high seroprevalence of bufavirus in the Middle East and Africa provides new opportunities for detecting bufavirus primary infections because these infections seem to be endemic to these regions. The predominant bufavirus genotype varied: bufavirus 1 was the most prevalent type in Finland and in the Middle East, and bufavirus 3 was the most prevalent type in the United States and in Kenya. Although IgG cross-reactivity was commonly observed, virus-specific antibodies could be distinguished from cross-reactivity by the competition assay. In contrast to bufavirus infections, cutavirus infections were distributed evenly and at found at low prevalences in all countries studied; for blood donors in the United States no virus IgG was detected. Tusavirus IgG was not detected in any cohorts studied.
